# An integrated-delivery-of-care approach to improve patient reported physical function and mental wellbeing after orthopedic trauma: study protocol for a randomized controlled trial

**DOI:** 10.1186/s13063-017-2430-5

**Published:** 2018-01-11

**Authors:** Laura Zdziarski-Horodyski, MaryBeth Horodyski, Kalia K. Sadasivan, Jennifer Hagen, Terrie Vasilopoulos, Matthew Patrick, Robert Guenther, Heather K. Vincent

**Affiliations:** 1 Departments of Orthopaedics and Rehabilitation, University of Florida, Gainesville, FL 32608 USA; 2 Departments of Anesthesia, University of Florida, Gainesville, FL 32608 USA; 3 Departments of Clinical Psychology, University of Florida, Gainesville, FL 32608 USA

**Keywords:** Orthopedic trauma, Psychosocial, Physical function, Emotional wellbeing

## Abstract

**Background:**

Orthopedic trauma injury impacts nearly 2.8 million people each year. Despite surgical improvements and excellent survivorship rates, many patients experience poor quality of life (QOL) outcomes years later. Psychological distress commonly occurs after injury. Distressed patients more frequently experience rehospitalizations, pain medication dependence, and low QOL. This study was developed to test whether an integrative care approach (IntCare; ten-step program of emotional support, education, customized resources, and medical care) was superior to usual care (UsCare). The primary aim was to assess patient functional QOL (objective and patient-reported outcomes) with secondary objectives encompassing emotional wellbeing and hospital outcomes. The primary outcome was the Lower Extremity Gain Scale score.

**Methods/design:**

A single-blinded, single-center, repeated measures, randomized controlled study is being conducted with 112 orthopedic trauma patients aged 18–85 years. Patients randomized to the IntCare group have completed or are receiving a guided ten-step support program during acute care and at follow-up outpatient visits. The UsCare group is being provided the standard of care. Patient-reported outcomes and objective functional measures are collected at the hospital and at weeks 2, 6, and 12 and months 6 and 12 post surgery. The main study outcomes are changes in Patient-Reported Outcomes Measurement Information System (PROMIS) questionnaires of Physical Function quality of life, Satisfaction with Social Roles, and Positive-Illness Impact, Post-Traumatic Stress Disorder Check List, and the Tampa Scale of Kinesiophobia-11 from baseline to month 12. Secondary outcomes are changes in objective functional measures of the Lower Extremity Gain Scale, handgrip strength, and range of motion of major joints from week 2 to month 12 post surgery. Clinical outcomes include hospital length of stay, medical complications, rehospitalizations, psychological measures, and use of pain medications. A mixed model repeated measures approach assesses the main effects of treatment and time on outcomes, as well as their interaction (treatment × time).

**Discussion:**

The results from this study will help determine whether an integrative care approach during recovery from traumatic orthopedic injury can improve the patient perceptions of physical function and emotional wellbeing compared to usual trauma care. Additionally, this study will assess the ability to reduce the incidence or severity of psychological distress and mitigate medical complications, readmissions, and reduction of QOL after injury.

**Trial registration:**

ClinicalTrials.gov, NCT02591472. Registered on 28 October 2015.

**Electronic supplementary material:**

The online version of this article 10.1186/s13063-017-2430-5) contains supplementary material, which is available to authorized users.

## Background

Trauma resulting in musculoskeletal injury is an unforeseen life-changing event. Nearly 2.8 million Americans sustain traumatic orthopedic injuries such as major fractures or amputation each year [1]. Severe injuries often require prolonged hospital stays with multiple reconstructive surgeries [2, 3]. Once the acuity of the injury is over, patients are left with the nebulous task of reintegrating into their lives. Although medical advances have dramatically improved survivorship, these injuries nevertheless result in poor quality of life (QOL)-related outcomes in otherwise healthy people [4]. Concomitantly, 50–90% of patients develop severe psychological distress such as post-traumatic stress disorder (PTSD), depression, or anxiety [5–7]. One exacerbating factor for this pattern is that patients are typically not provided comprehensive support and resources that are necessary to successfully cope with psychological distress [8]. This is a serious issue because high distress levels predict poor physical function, use of pain medications and low QOL [9, 10]. Trauma survivors often cannot return to work, [11] have persistent pain [12], and experience social isolation. Distress worsens the self-perceptions of functional gain and efficacy [13] and decreases personal fulfillment. Lingering psychological distress contributes to the development of other health problems [14, 15]. The lack of psychosocial support contributes to injury reoccurrence, injury recidivism, [16] rehospitalizations and longer hospitalization, [17] and higher personal and societal healthcare costs [18].

Development of programs that can help reduce psychological distress and provide focus to patients may help fully engage patients in therapeutic activity and ease the transition from hospital to home. There is currently a lack of rigorous comparative efficacy trials to determine whether programs like this can impact functional QOL and emotional wellbeing. Currently, usual trauma care focuses on the medical and anatomical restoration of the patient. It does not, however, provide the simultaneous psychosocial and emotional support that patients need early in the care process to cope with their injuries, stress, and understand the recovery process. This communication and support gap in care worsens the psychopathology of orthopedic trauma. The patient, while receiving the latest medical care for their injuries, does not receive the overall care needed to treat the entire person. An integrative care approach, which involves a facilitator-driven ten-step support program, may help patients develop focus, engage in the recovery process, and set up supportive networks before leaving the hospital. In so doing, trauma survivors may better cope with the hardships after hospital discharge and into their recovery process.

## Methods

### Objectives

#### Primary objective

The primary objective is to determine if IntCare (integrated care) improves functional QOL better than UsCare (usual care) in patients receiving care for an orthopedic trauma injury.

#### Secondary objectives

Secondary objectives include: (1) determining whether IntCare improves markers of emotional wellbeing more than UsCare; and (2) comparing the prevalence of medical complications, rehospitalizations, and co-morbid disease up to 12 months after surgery.

First, we hypothesize that IntCare will improve functional QOL and emotional wellbeing after hospital discharge more than UsCare [8, 19, 20]. Second, we hypothesize that patients with IntCare will have fewer rehospitalizations, medical complications, and co-morbid psychological illness compared to patients with UsCare.

### Design

**Fig. 1:**
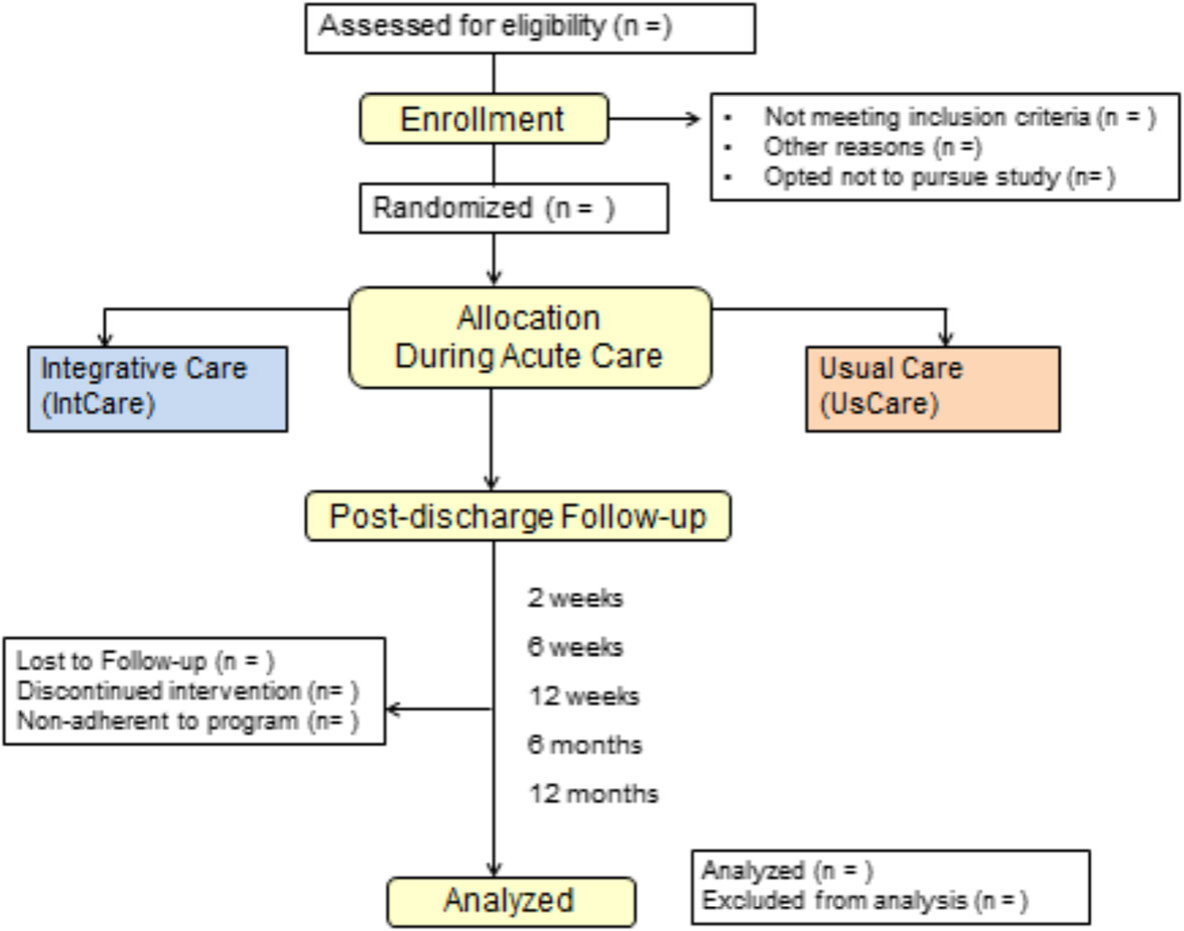
Study *flow diagram* following the CONSORT guidelines for randomized controlled trials with the Patient-Reported Outcomes extension

This is a single-center, single-blinded, repeated measures, randomized exploratory controlled study with parallel 1:1 allocation in which the research and care teams, including the physicians, know which patients are receiving the integrated medical care or usual medical care [21]. Figure 1 provides the study flow diagram of this ongoing study, for which there are two study arms: the IntCare arm and UsCare arm. We are executing this study under the Consolidated Standards of Reporting Trials (CONSORT) Statement [21] for randomized controlled trials with the Patient-Reported Outcomes extension [22]. This is an Investigator Initiated Trial that was registered with ClinicalTrials.gov (NCT02591472) on 28 October 2015, before patient enrollment was initiated. The Institutional Review Board for the Protection of Human Subjects at the University of Florida approved all study procedures. The project was launched in November 2015. The trial is financially supported in part by the Foundation for Physical Medicine & Rehabilitation (internal funding reference number 00098192), the National Athletic Trainers’ Association Research & Education Foundation (grant number 15DGP012), and the W. Martin Smith Interdisciplinary Patient Quality and Safety Award. The trial meets the criteria described in the Standard Protocol Item: Recommendations for Interventional Trials (SPIRIT) checklist (Additional file 1).

### Patients

#### Inclusion criteria

The inclusion criteria were as follows: aged 18–85 years; admitted with severe or multiple orthopedic trauma (any major bone fractures that impairs mobility and/or participation in activities of daily living and self-care); and have received or will receive one or more surgical procedure for their orthopedic injuries.

#### Exclusion criteria

The exclusion criteria were: presence of a traumatic brain injury; inability to communicate effectively (e.g. at a level where self-report measures could not be answered completely such as medicated state or mechanically ventilated); currently using psychotropic medications; or have psychotic, suicidal, or homicidal ideations. All participants provided written consent on an institution approved consent document.

### Recruitment

Study recruitment began in November 2015. Patients were initially approached by their orthopedic trauma physician after admission to the Orthopedic Trauma service at UF Health at Shands Hospital in conjunction with the University of Florida (UF). To ensure adequate recruitment, the trauma physicians screened all persons admitted to their care.

### Registration and consent

Patients were medically stable before initiation of recruitment by the physician and/or the research team. The patients’ orthopedic trauma physician provided patients and family (as appropriate) with a brief overview and explanation as to why they were conducting the trial. If a patient expressed interest in participating, study personnel then met with the patient to cover all study-related information and to address questions the patent or their family may have about study participation. The physician and study team made a concerted effort to explain the voluntary nature of the study and that their decision to participate would have no impact on their medical care. Patients were informed that at any time they could withdraw themselves from the study as well as the study staff could with draw them if it became apparent continued participation was not in their best interest.

A signed and dated copy of the consent form was provided to participants. A second signed and dated copy of the consent form was retained by the study staff. These records are stored in a locked file cabinet and locked office within the lab located in the University of Florida Orthopedic and Sports Medicine Institute, the outpatient facility.

### Locations

UF Health at Shands Hospital is located Gainesville, Florida, USA. This is a level 1 trauma center with a catchment area of 18 urban and rural counties comprising two million people. Approximately 2500 patients with traumatic injuries are admitted annually. Consent and baseline data were collected at the level 1 trauma center. After discharge, patients are followed up with at the University of Florida Orthopedic and Sports Medicine Institute. This facility houses the outpatient orthopedic clinics and is where all patients receive their follow-up care.

### Trial intervention

Dr. Sadasivan’s previous work with a doctoral student in psychology revealed the strong need for psychosocial care in acute care after orthopedic trauma [8]. A subsequent pilot study was conducted to evaluate feasibility of performing research with orthopedic trauma patients in the acute setting and the outpatient clinic. The present trial is the result of many years of working with orthopedic trauma patients and identifying the need for more comprehensive care and developing work flow patterns. The pilot study occurred over the course of one year and with follow-up up to three months and only a select number of survey instruments utilized. The State-Trait Anxiety Inventory (STAI) and Beck Depression Inventory-II (BDI-II) were used in the pilot and were then used to help conduct our power analysis for the present trial design. During the course of the pilot study, our research group relied on our institutional Orthopaedic Trauma Patient Advisory Panel for input on patient needs and appropriate ways to measure better outcomes. Dr. Vincent used the panel’s input to create the interactive folder. Dr. Zdziarski-Horodyski developed a resource manual, such as pet care services, for patients (Table 1).

**Table 1:** Excerpts from the manual containing resources that can be recommended to trauma patients by the study facilitators. The content of the resource manual should be specific to the geographic location of the trauma center

*Job placement agencies*
Vocational rehabilitation (Florida Division of Vocational Rehabilitation) Our Mission is “to help people with disabilities find and maintain employment and enhance their independence.” Our Vision is “to become the first place people with disabilities turn when seeking employment and a top resource for employers in need of qualified employees.” http://www.rehabworks.org/ Contact information: 352-955-3200 – Gainesville location 2610 NW 43rd St Suite 1A Gainesville, FL 32606; 800-451-4327 – Toll-free State Office; 850-245-3399 – Tallahassee State Office
*Home modifications*
Christian Concerned for the Community. Ramps, shower bars; (352)-371-1768; http://cccgainesville.org/
Center for Independent Living. Mission “The CILNCF is an established community disability resource center operated by people with disabilities and serving North Central Florida for over 30 years. We deliver high quality programs and services that enhance quality of life and increase levels of personal independence.” Housing/Repairs – can help build ramps. Contact information: 1-800-265-5724
*Transportation and food*
Regional Transit System (RTS). Services the Gainesville city limits; most buses are accessible for all disabilities (please see website for routes and specifics http://go-rts.com/ada/). Bus fares are in the range of $0.75–1.50/each way (see website for details)
Eldercare of Alachua County. Utilities/rent assistance, transportation assistance, food services (meals on wheels and private pay meal plan), homemaking, personal care, and respite. Contact information: (352)-265-9040; http://eldercare.ufhealth.org/about-eldercare/contact-eldercare/
Ride Solution. Palatka, FL. Can assist with transportation to and from Palatka, FL to Gainesville, FL. See website for bus stop locations and scheduling information. http://theridesolution.org/# $2.00 bus fare
Meals on Wheels. In partnership with ElderCare of Alachua County. Providing meals to the elderly who are in need. http://eldercare.ufhealth.org/services/meals-on-wheels. Gainesville – Thelma Boltin Center. Contact information: (352)-334-2189, 516 NE 2nd Avenue, Hours: 10 am – 1 pm, Monday – Friday
*Pet services*
Daytime Dogs and Friends. Mission: “Our goal is to deliver convenient, personalized, reliable services in a caring and trustworthy manner to all of our clients and their beloved pets.” Contact information: 352-219-4246. http://www.daytimedogs.com/

#### Usual care (UsCare)

Postoperative care is based on widely accepted recommendations [8] and on the current understanding of injury treatment. The key components of UsCare include medical stabilization, injury repair, discharge planning, and acute care therapies. Participants randomized to the UsCare group receive medical care from a standard orthopedic medical team without the presence of a facilitator. All other routine care from the physical therapist and discharge planner occur as would usually transpire at all level one trauma facilities across the United States. Baseline measurements for all study instruments (described in the next section) are administered after consent and at the subsequent follow-up appointments at the Orthopaedic and Sports Medicine Institute by the research team. At the participant’s 12-month follow-up visit, all educational materials are provided and a single meeting with one of the facilitators to go over the “Transform 10” is offered.

#### Integrated care (IntCare)

**Fig. 2:**
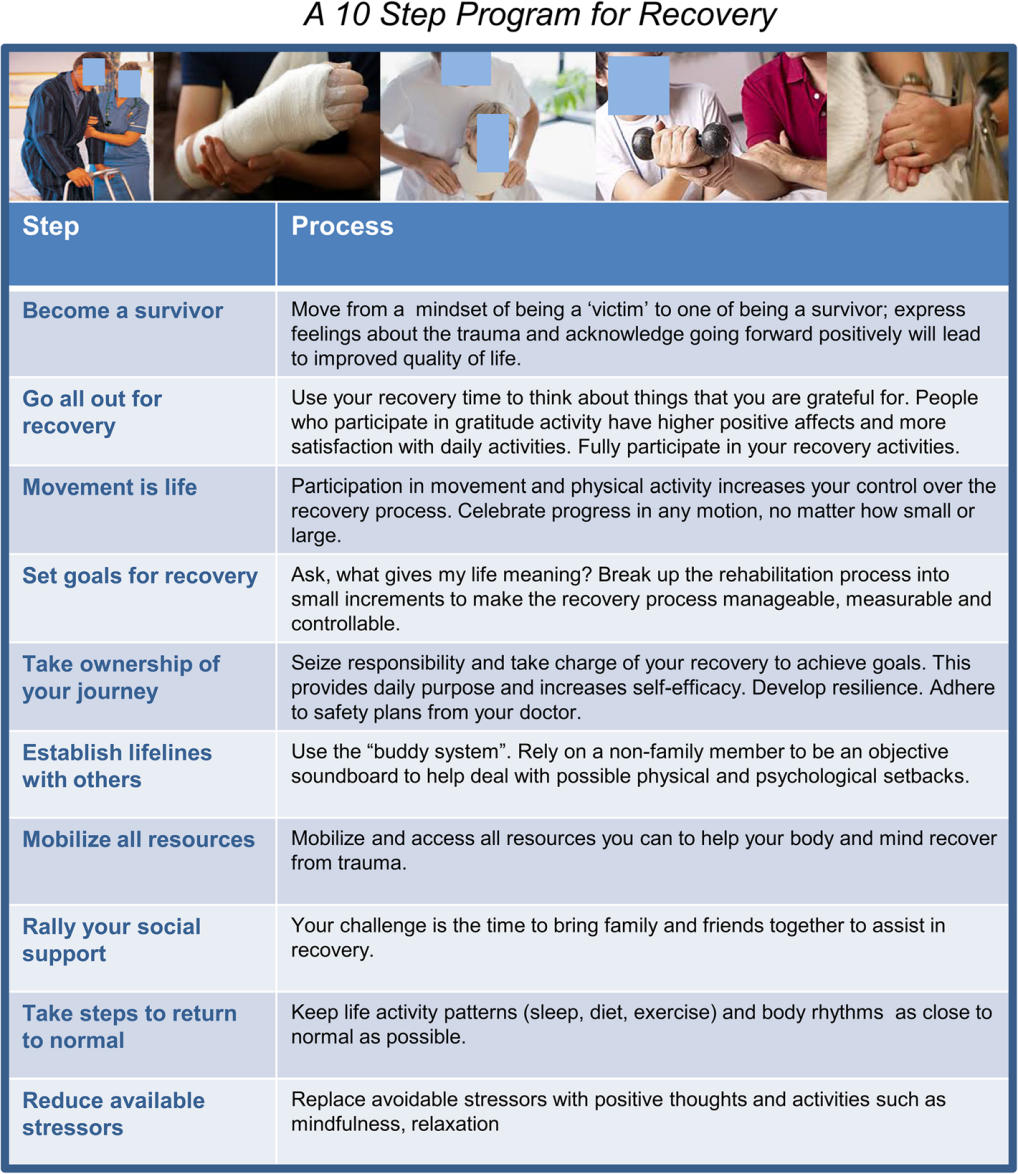
The ten steps of the Transformative Coaching Program. The order of the steps may be adjusted as needed based on the status of the patient

IntCare provides all UsCare processes as described above, plus psychosocial support via a ten-step transformative program (“Transform 10”). Psychosocial components and resource content are included to help patients focus on the positive and productive pathways necessary to cope with stress and achieve a high QOL. Facilitators help patients identify the immediate concerns and help identify solutions to address them. Common concerns include loss of job, child care, obtaining food or medicine, transportation to appointments, and wheelchair access to the home environment. The steps of this program are shown in Fig. 2 [23–32]. Key components can be adapted for different hospital settings, geographical locations, and available resources when implemented in a larger scale. Facilitators help patients develop productive focus on the short-term goals (e.g. acknowledging that they are a survivor, developing the mindset to move forward with surgeries, and rehabilitation that will help improve their physical status, decrease stressors, and set goals for recovery) and long-term goals (e.g. be wheelchair-independent by month 3, able to pick up child on own by month 4, get back to work as fast as possible). The program provides information and customized resources needed to help patients empower themselves to achieve these goals, optimize physical and mental health, and develop resilience.

**Fig. 3:**
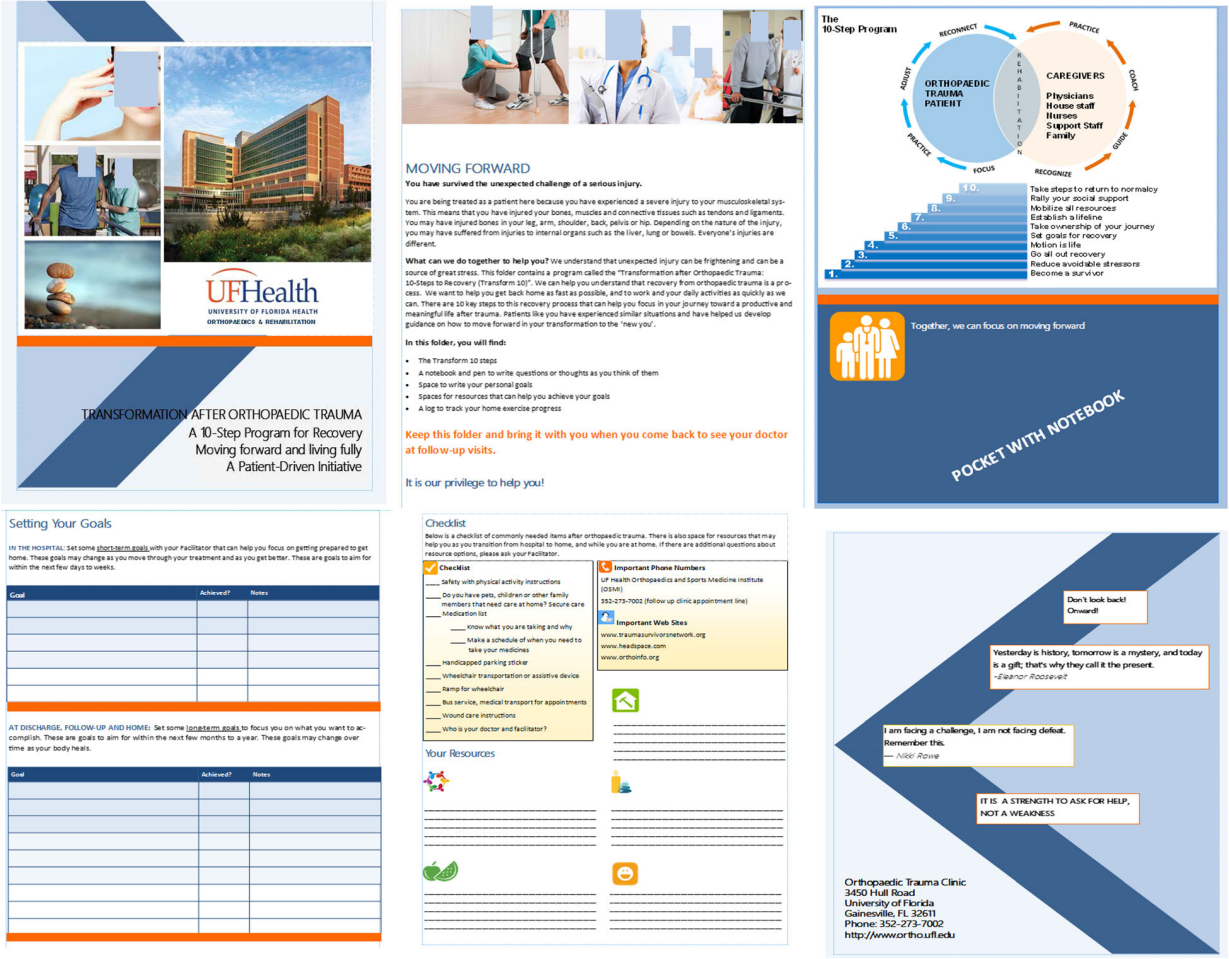
Images of trifold patient folder containing the 10-step program, goal setting space, pre-discharge checklist, space for customized resources and space for paper and pen

The facilitator provides a folder containing the “Transform 10” program, pen and notepad, and spaces to record appropriate resources that the patient believes would be helpful for them. A view of the opened patient folder is shown in Fig. 3. The study team compiled a large regional resource list; a sample is shown in Table 1. Specific resources are recommended to patients depending on their needs. Lastly, patients within this group receive a structured, physician-approved exercise program at follow-up visits to promote movement and strength before beginning supervised physical therapy. While the patient is hospitalized, daily interactions occur with patients by facilitators to promote the “Transform 10” steps and when needed provide information to the attending orthopedic surgeon. As many steps as possible are initiated with each patient during acute care. Before beginning new steps, previous steps are reviewed. At the outpatient follow-up visits, the patient is asked to bring their folder to review any steps as needed and present any steps that were not presented in acute care. Facilitators help patients self-manage any confounding issues or barriers to reaching their goals.

#### Facilitators

All facilitators utilized in this trial had at minimum a college level degree in a health-related field. The two primary facilitators were athletic trainers, one with a EdD in Kinesiology and the second a PhDc in Rehabilitation Sciences. The other facilitators had varying levels of training from bachelors in Sport Psychology to masters in Health and Human Performance. The two primary facilitators received training and oversight from Dr. Sadasivan and the clinical psychologist (mainly in how to refer patients in destress, see “Distress referral protocol”), as well as played an integral role in creating training materials for future facilitators. All other facilitators first were interviewed to assess their abilities and desire to work with this population. Next, facilitators were provided the scientific literature for each of the ten steps, had an observation period with one of the two primary facilitators, practiced scenarios, and supervised intervention administration before administering the intervention on their own. Additionally, every week the entire study team (facilitators, researchers, and physicians) would meet and discuss any difficulties encountered earlier in the week or provide feedback on various observations of the intervention. Every IntCare patient was progressed through all ten steps; however, every patients’ needs are different and therefore the specifics discussed under each step may be different. The weekly meetings allow for continuous facilitator development.

The facilitators were also responsible for encouraging and monitoring adherence to the intervention. This is achieved through the interactive folder provided to the patients where goals and other needs can be recorded. Facilitators recorded the patient’s goals in the associated study folder as well as any other notes as they found necessary when covering a step, so that when the patient returns for follow-up visits accountability can be achieved. All of this information is recorded on data collection forms within the study folders.

### Randomization

#### Allocation and concealment

All eligible participants were consecutively randomized to either the IntCare or UsCare groups. Randomization process was conducted using a computer-generated random number list and consecutively numbered opaque envelopes containing the group allocation. These were prepared by a Clinical Coordinator not involved in the testing. Every study participant’s folder was prefilled by the Clinical Coordinator with all materials for the study, randomization envelope, consent forms, supplemental materials, and all data collection forms regardless of group assignment. This ensured that study staff would not know which group the patient would be assigned to before opening the envelope.

### Blinding

Patients were blinded to which treatment group they were randomized. Study staff opened the randomization envelope in the study office at the hospital, outside of the patient’s room after obtaining consent. The patient was never explicitly told to which group they had been assigned. After the research staff members opened the envelope, they promptly returned to the patient’s room with the iPad and other data collection materials to begin data collection.

### Data collection

The study team is following a systematic process for data collection using electronic case report forms (CRFs) that follow *Good Clinical Practice* rules. Electronic CRFs are managed using the Research Electronic Data Capture (REDCap) [33]. Data are validated at the time of input by computerized controls that ensure validity and quality. REDCap contains system integrity measures to guarantee the integrity of the system and to protect against data loss. On a weekly basis, all records are reviewed by the study team. All participant data is de-identified and stored under a consecutively numbered study code. Consent forms are the only document that have patient names and are immediately removed from the study folder after signing. The consent forms are then stored in the study binder locked in the PI’s office; no study code is associated with the consent forms. All study-related data and materials will be kept the minimum number of years after study closure, per government standards, in a locked cabinet and incinerated after the period of time.

Per several of the funding mechanisms, quarterly reports on trial progress are generated and serve as an internal data monitoring process. No external agency will monitor the data, outside the University’s IRB if necessary. The study team and physicians will have access to the quarterly report generated, should anything in the data suggest that the trial be stopped the physicians will be able to provide judgement.

#### Patient timeline

Baseline measurements for all patient-reported outcomes, measures of psychological distress, and handgrip strength were collected while the patient was receiving acute care. After discharge from acute care, patients return to the outpatient orthopedic trauma clinic for regular follow-ups. The study team is minimizing the patient burden by collecting data at the normal outpatient visits which occur at weeks 2, 6, and 12 and at months 6 and 12 post surgery. Patients were asked for email addresses after consenting so that surveys may be emailed, if the patient prefers and based upon patients’ resources. Therefore, patients may complete the surveys at home before their follow-up visits. For patients not using email, study staff may conduct the surveys over the phone if the patient indicated the desire. If surveys are not completed before their visit, the patient will be met once they have checked in and then provided an iPad to complete the electronic surveys. All functional measures are obtained in the Human Dynamics Laboratory, located on the first floor of the outpatient clinic.

**Fig. 4:**
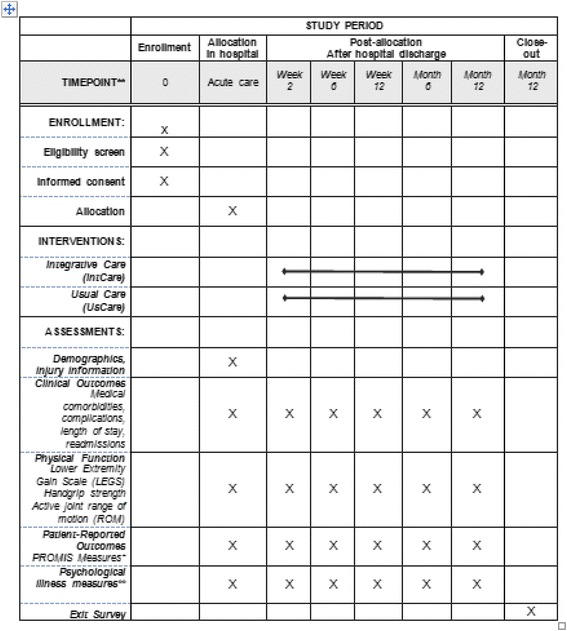
Schedule of study enrollment, interventions, and assessments

At the 12-month follow-up (in some special cases where the patient’s orthopedic care was complete, at the six-month follow-up) an exit survey is given. This survey aims to understand the patient’s experiences throughout the study and their care. The study team recognizes that patient responses may be influenced by their perception of care given. Additionally, the survey will help ensure no cross-contamination of study participants. A majority of orthopedic trauma patients are cared for on the same floor; therefore, patients have the potential to interact with each other in the common spaces (all patient rooms are single occupancy). At this 12-month time point, the UsCare group is notified as to their randomization. Individuals assigned to this group will be given the opportunity to receive all the materials the IntCare group received. The study schedule overview is shown in Fig. 4.

## Outcome measures

### Primary outcome measures

#### Physical function

Objective clinical measures of physical function complement the patient-reported outcomes. Three key measures are being collected: the Lower Extremity Gain Scale (LEGS); handgrip strength; and joint range of motion (ROM) [34]. LEGS was the primary outcome of this study. First, the LEGS assessment consists of several lower body movements that occur in daily life [35], including a 3-m walk, putting on a sock, putting on a shoe, rising from an armless chair, stepping up and down stairs, getting on and off the commode, and reaching from a sitting position to an object on the ground. In people with traumatic fractures, LEGS has high internal consistency and the content, concurrent, and construct validity are high [35]. The clinical relevance of better physical function and ambulation scores is a reduced risk of infection, delirium, and prolonged hospital stay [36]. Second, isometric handgrip strength is a valid predictor of mobility and QOL and is being measured using a hand-held hydraulic dynamometer [37]. Handgrip strength is clinically important as it strongly predicts long-term function capability after orthopedic trauma [37]. The intraclass coefficient (ICC) for handgrip strength testing is 0.95. Third, the use of active ROM (AROM) as a measure of functionality is common across multiple disciplines, including orthopedics, physical therapy, and athletic training. Establishing early and appropriate AROM within and at the joints above and below the injury site in the subacute/pre-structured physical therapy phase is significantly correlated [38] with increased functional outcomes [39]. AROM is being measured with a goniometer and a digital inclinometer [40]. Lower extremity ROM will be collected for hip flexion, knee flexion/extension, and ankle plantar/dorsiflexion. Upper extremity ROM joints will include: shoulder flexion/extension, abduction, and internal/external rotation; elbow flexion/extension; and wrist flexion/extension.

### Secondary outcome measures

#### Patient-reported outcomes

Patient-reported outcomes are the primary measures of the study. Patients are the most important source of information regarding the outcomes of interest, because this study focuses on patient perceptions of functional QOL and emotional wellbeing. Patient-Reported Outcomes Measurement Information System (PROMIS™) assessments are administered using computer adaptive tests [41]. Testing of PROMIS™ domains has been performed in patients with various upper and lower body orthopedic injuries [42, 43]. As indicators of functional QOL and emotional wellbeing, the PROMIS measures of Physical Function, Psychosocial Illness Impact Positive, and Satisfaction with Social Roles and Activities are being administered.

#### Clinical outcomes

Electronic medical records are being used to obtain information on patients, including sociodemographic and socioeconomic characteristics, trauma injury type and severity, location, and additional soft-tissue injuries. Information about the nature of the trauma is being obtained including issues that may have precipitated their orthopedic injury (e.g. drunk driving, drug use, if other individuals were injured/ killed in the accident). The prevalence of medical complications, rehospitalizations, and co-morbid disease is also being captured in two ways: (1) using data extraction methods from electronic medical records; and (2) directly from patients during their follow-up visits in the outpatient clinics. The number of readmissions (and length of the readmissions), reasons for readmissions, the number and type of complications are being collected.

The onset of new co-morbid diseases, with particular emphasis on psychological illnesses, is also being collected using these following tools that have been validated for use in the trauma population. These include the PTSD Checklist, with high temporal and internal consistency and high content validity [44], the BDI-II, with high reliability and consistency [45], and the STAI, with high internal consistency in the range of 0.86–0.95, and construct and concurrent validity [46]. Kinesiophobia is the psychosocial, somatosensory neuronal feedback, manifestation of fear of movement due a belief it will induce pain or injury [47, 48]. To assess the pain-related fear in orthopedic trauma the Tampa Scale of Kinesiophobia-11 in being used, with an interclass correlation of 0.81 [48].

#### Distress referral protocol

We acknowledge that for some patients the level of psychological distress is beyond the capability of this program to provide the support necessary. In these cases, we will enact a distress referral protocol. Patients, who have BDI-II, STAI, and/or PTSD scores > 2 standard deviations (SD) from the population norm will be referred for further evaluation by the Psychology Service via the electronic medical record (EPIC) referral process. Those services will include a formal clinical interview, development of a diagnostic conceptualization, development of a treatment plan, and provision of treatment designed to reduce the patient’s distress and improve coping. Treatment will usually consist of cognitive-behavioral interventions with strong scientific support for their efficacy. Additionally, if the patient answers the STAI question about suicide with any response other than, “I do not have any thoughts of killing myself,” their physician is immediately notified to further assess the situation.

### Analysis

#### Sample size

A sample size of 100 was determined in an a priori manner using the G*Power software program [49, 50]. Anticipating that the study population will be younger but otherwise similarly distributed as that of Zimmerman et al. [35], the sample size, n = 100, was determined to be sufficient to have a medium effect size (Cohen’s d = 0.60, power of 0.80, and alpha of 0.05). Data from Zimmerman et al.’s [35] study validating the established the LEGS was used as the primary measure to power for the study. This analysis then translates into variable detectable mean differences depending on the outcome. For example, a 6.4-point difference in the STAI (one of the psychological measures for the study) can be detected assuming a SD of 10.0; for AROM, 8.3° difference with a 12.0° SD. This sample size is therefore expected to be sufficient to determine if differences in functional QOL and emotional wellbeing occurred.

#### Statistical analysis

The Statistical package for the Social Sciences (SPSS, v 24.0; Chicago, IL, USA) will be used for analysis. Descriptive statistics will be calculated on categorical study variables and demographics (means and SD for continuous variables, frequencies and percentages for categorical variables). Chi-square for frequency distributions will be used for patient satisfaction to test main effects of time and treatment and their interaction. The primary analyses for all aims will utilize a mixed model repeated measures approach. These analyses can assess the main effects of treatment and time on outcomes, as well as their interaction (treatment × time). Specifically, independent variables will include care approach (integrated vs standard) and time point (baseline, weeks 2, 6, 12, months 6 and 12 post surgery). Dependent variables will include all PROMIS™ and functional measures. Mixed models are the preferred approach to analyze data with repeated measures; these models can account to for correlation among repeated measurements, flexible time effects, and can handle missing data. Significant interactions between treatment and time would indicate that the change in the outcomes (i.e. slope) was dependent on the patient’s treatment group. If a significant interaction is identified, the Preacher method will be used to estimate the magnitude and direction of the interaction. A *p* value will be established a priori at < 0.05 for all statistical tests. Continuous data that are not normally distributed will be transformed before analysis. Appropriate multiple testing corrections will be performed to limit Type I errors.

## Discussion

At present, there is a lack of rigorous, high-quality, comparative effectiveness research to determine whether a comprehensive care approach for both physical and emotional health produces greater improvements in the key outcomes functional QOL and emotional wellbeing. This study tests the hypotheses that IntCare can improve patient-reported outcomes of functional QOL and emotional wellbeing more than UsCare, and that IntCare will result in fewer medical complications and hospital readmissions, and a lower incidence of psychological illness onset in patients recovering from orthopedic trauma. A combination of subjective and objective assessments is being used to test these hypotheses.

While some psychosocial support is typically available in the hospital, it is often reserved for patients with acute crisis or if deemed a threat to themselves or others. As such, psychosocial support is not typically addressed at all during acute trauma care. Support is more likely obtained by patient’s months after they are at home trying to cope with the adjustment to life. The Trauma Survivors Network (TSN) and other research groups have proposed steps to provide IntCare systems in real-life settings [51, 52]. The TSN is a public-health approach (consisting of peer support, self-management help, information, and resources) that was designed to help trauma patients overcome challenges in recovery and increase self-efficacy, functional outcomes, and wellbeing. Limited evidence has revealed significant improvements in perceived health and lower rates of depression in the TSN service group compared to UsCare [4]. Early IntCare can empower the patients to become resilient, active participants in their care. A smaller randomized controlled trial showed that an early focus on patient motivation with aspects of psychosocial support reduces the length of hospital stay and improves the trajectory of recovery after hip fracture [53]. Compared to patients with high stress, patients with low stress levels achieved greater functional status levels and resilience in recovery [52]. The use of various psychosocial support such as counseling, pastoral care, coping skills for pain, meditation, and mindfulness can reduce patient anxiety and depression at one month post discharge by 16–66% [54]. Support intervention compared to usual care improved QOL domains of physical function, vitality, physical role limitation, and mental health by 34–95% and reduced the need for pain medications in people with hip fracture [55]. Early administration of an educational intervention (breathing exercise, education on pain management, relaxation techniques) reduced anxiety, improved self-efficacy, and reduced hospital length of stay by 20% compared to UsCare in patients with various trauma injuries [56].

The use of facilitators with non-mental healthcare backgrounds is a novel method of providing patients emotional support, patient education, focus, and a connection to resources that may help patients reach their recovery goals. Hence, this study will help determine if support provided by facilitators with allied health training, non-psychology trained personnel, can positively impact patient outcomes. This will add to the feasibility of developing these intervention models in other healthcare systems and will empower orthopedic care teams by giving them a toolkit that does not rely heavily on mental healthcare services. The evidence generated from this study will help provide future framework to better empower patients to participate more effectively in their care and recovery and achievement of personal goals for recovery. This research should assist clinicians and healthcare system managers to make informed decisions about implementation of a system that produces the best outcomes for their patients.

### Potential limitations

Potential limitations to the present trial design include interference with the control group and a stringent monitoring policy for intervention adherence. While the research team has taken very cautious measures with a control group, it has become apparent that the simple interaction of asking orthopedic trauma patients to complete surveys about how they are feeling and functional tests may give the perception that they are receiving additional “care.” The research team recognizes that the interactions to collect data from the control group at present are necessary and unavoidable, but may be positively influencing this group’s outcomes. In future study design, a control group with limited time points for follow-up could be utilized. Patients agreeing to participate in this trial are not receiving clinical mental healthcare and their participation is strictly voluntary. Therefore, ensuring strict adherence to the intervention may be a limitation to the trial design. The use of the interactive folder is one mechanism in place to address adherence; however, participants do not receive compensation and thus repercussion for non-adherence is not feasible.

### Dissemination plans

The authors plan to publish a manuscript focusing on the primary and secondary objectives in the *Journal of Orthopaedic Trauma* at the completion of the trial. Furthermore, data will be presented at various conferences in an effort to target all areas of healthcare. In the immediate future, data will be shared and the themes learned from the trial communicated with the house staff within the university hospital.

### Trial status

Enrollment of patients was initiated in November of 2015 and data collection and analyses are expected to be completed in November of 2018. Data collection is ongoing.

## Additional file

Additional file 1: SPIRIT 2013 Checklist: Recommended items to address in a clinical trial protocol and related documents. (DOC 120 kb)

## Abbreviations

AROM: Active range of motion
BDI-II: Beck Depression Inventory second version
ICC: Intraclass correlation coefficient
IntCare: Integrated care treatment group
LEGS: Lower Extremity Gain Scale
PhDc: Doctorate of Philosophy Candidate (a term used in the United States higher education system to indicate that a PhD student has passed their qualifying exams and successfully proposed their dissertation topic)
PROMIS: Patient Reported Outcome Measurement Information System
PTSD: Post-traumatic stress disorder
QOL: Quality of life
SD: Standard deviation
SPSS: Statistical Package for the Social Sciences
STAI: State Trait Anxiety Inventory
TNS: Trauma Survivors Network
UsCare: Usual care treatment group
